# Job satisfaction of nurses and identifying factors of job satisfaction in Slovenian Hospitals

**DOI:** 10.3325/cmj.2012.53.263

**Published:** 2012-06

**Authors:** Mateja Lorber, Brigita Skela Savič

**Affiliations:** 1 University of Maribor, Faculty of Health Sciences, Maribor, Slovenia; 2 Dean of College of Nursing Jesenice, Faculty of Management Koper, University of Primorska, Koper, Slovenia

## Abstract

**Aim:**

To determine the level of job satisfaction of nursing professionals in Slovenian hospitals and factors influencing job satisfaction in nursing.

**Methods:**

The study included 4 hospitals selected from the hospital list comprising 26 hospitals in Slovenia. The employees of these hospitals represent 29.8% and 509 employees included in the study represent 6% of all employees in nursing in Slovenian hospitals. One structured survey questionnaire was administered to the leaders and the other to employees, both consisting 154 items evaluated on a 5 point Likert-type scale. We examined the correlation between independent variables (age, number of years of employment, behavior of leaders, personal characteristics of leaders, and managerial competencies of leaders) and the dependent variable (job satisfaction – satisfaction with the work, coworkers, management, pay, etc) by applying correlation analysis and multivariate regression analysis. In addition, factor analysis was used to establish characteristic components of the variables measured.

**Results:**

We found a medium level of job satisfaction in both leaders (3.49 ± 0.5) and employees (3.19 ± 0.6), however, there was a significant difference between their estimates (*t* = 3.237; *P* = <0.001). Job satisfaction was explained by age (*P* < 0.05; β = 0.091), years of employment (*P* < 0.05; β = 0.193), personal characteristics of leaders (*P* < 0.001; β = 0.158), and managerial competencies of leaders (*P* < 0.000; β = 0.634) in 46% of cases. The factor analysis yielded four factors explaining 64% of the total job satisfaction variance.

**Conclusion:**

Satisfied employees play a crucial role in an organization’s success, so health care organizations must be aware of the importance of employees’ job satisfaction. It is recommended to monitor employees’ job satisfaction levels on an annual basis.

Job satisfaction is determined by a comparison of one’s prior expectations about the job and the actual experience of the job (1). It has been found that job satisfaction relates to beliefs and emotions that individuals have about their work and their job (2). It has been described as an attitude with an affective and cognitive component (3). When establishing the level of job satisfaction, we should focus on how employees feel about their work and personal relationships in the workplace, and on how leaders influence employees’ satisfaction. Without a doubt, satisfied employees are the ultimate goal of every leader. On the other hand, the goal of every employee is to find the kind of work that matches their abilities and interests as closely as possible, enables them success, and provides them with opportunities for promotion. Satisfied employees tend to be more productive and committed to their employers, and a direct correlation has been shown between staff satisfaction and patient satisfaction in health care organizations (4,5).

Even though research has shown different levels of job satisfaction for nurses, satisfaction predictors tend to be relatively similar, and include working conditions, relationships with coworkers and leaders, pay, promotion, security of employment, responsibility, and working hours (2,6-16). In Slovenia, no studies on the effects of leadership style, personality characteristics, and managerial competencies of leaders on job satisfaction have been conducted. There was only some research about leadership style in health care institutions (17-19). Also organizational climate and organizational culture in nursing have been studied as well as job satisfaction in some institutions (20-24). Experts in Slovenia (17,18,25-27) point to the problem of lack of knowledge of leaders about leadership.

Because it affects not only quality of nursing but also patients’ satisfaction, the level of employees’ job satisfaction is very important for health care institutions. The aim of the study was to determine the level of job satisfaction of employees in nursing and to determine the influence of leadership in job satisfaction.

## Methods

### Sample and study design

Study took place in 2009 in 4 major Slovenian hospitals – University Clinical Center Maribor, General Hospital Celje, General Hospital Slovenj Gradec, and General hospital Murska Sobota. Five major Slovenian hospitals have been selected from the hospital list, but 1 refused to participate. Employees in the 4 participating hospitals represented 29.8% of employees in nursing in all Slovenian hospitals. The questionnaires (web-extra material) (web extra material 1) were distributed in the morning shift, by authors in one hospital and by research coordinators in other 3 hospitals. There were 750 questionnaires distributed, which amounts to 26.8% of 2802 employees in nursing in Slovenian hospitals that participated in the study and 8% of 9404 employees in nursing in all Slovenian hospitals. Hundred and ten questionnaires were sent to middle- and unit-level nurse leaders and 640 to other nursing employees. Nurse leaders were not selected randomly; the questionnaires were sent only to those who occupied the position of head of department, unit, or clinic, which means that purposive sampling was used. The maximum time for filling out the questionnaires was 14 days. Questionnaires were collected in specially designed boxes to ensure anonymity. Five hundred and nine questionnaires were correctly and completely filled out and the response rate was 68%. This sample represented 6% of all employees in nursing in Slovenian hospitals. The 4 hospitals had provided a written permission for research.

On the list of the Institute of Public Health of Republic of Slovenia, there are 26 hospitals. In the study in 2009 we wanted to include 5 (20%) of the 26 hospitals, so we asked every fifth hospital to participate and one of them refused. The other four hospitals employed 29.8% of all employees in nursing in Slovenian hospitals. Seven hundred and fifty questionnaires were distributed in the morning shift. Hundred and ten questionnaires were sent to middle and unit-level nurse leaders and 640 were sent to other employees in nursing. To ensure anonymity, questionnaires’ were collected in specially designed boxes. The response rate was 68%, because 509 questionnaires were completely filled out. Before the research, we obtained a written permission from the participating hospitals. This manuscript is part of a larger research, which has in part already been published (28).

### Instruments

Two survey questionnaires with 154 closed-type items each were used, one for leaders and one for other employees in nursing. The questionnaires were prepared based on the literature on modern leadership and managerial competencies of nursing leaders and in cooperation with the O.K. Consulting (company for education and research of employees in all areas), and had been tested in a pilot study (10 leaders and 30 employees). Leaders self-assessed their leadership style, managerial competencies, and characteristics on a 5-point Likert-type scale ranging from 1 (strongly disagree) to 5 (strongly agree). Employees assessed the leadership style, managerial competencies, and characteristics of their immediate superior on a 5-point Likert-type scale ranging from 1 (strongly disagree) to 5 (strongly agree). The first part of the questionnaire included demographic data: sex, age, institution, years of employment, years of employment in a leading position, and the level of education.

In the study we used two questionnaires, one for leaders and one for other employees in nursing. The questionnaires were prepared in the cooperation with the O.K. Consulting (Company for education and research of employees) and also based on the literature on leadership and job satisfaction. We used 5-point Likert-type scale ranging from 1 to 5, with 1 meaning strongly disagree and 5 meaning strongly agree (29). Leaders evaluated themselves, while other employees evaluated their immediate superior. Both, leaders and employees evaluated their own level of job satisfaction.

The first part of the questionnaire included demographic data: sex, age, institution, years of employment, years of employment in the leading position, and the level of education. The second part of questionnaire was prepared after an overview of relevant literature on modern leadership and managerial competencies of nursing leaders (15,30-33). This part of the questionnaire contains items on managerial competencies and leadership style. Cronbach α was 0.798. In the third part of questionnaire, nursing leaders and nurses indicated their job satisfaction levels, which reflected the relevant theoretical background in the field (2,12,34,35). Cronbach α was 0.849.

### Statistical analysis

The survey was based on quantitative methodology. For statistical analysis we used statistical program SPSS 16.0 (SPSS Inc., Chicago, IL, USA). Differences between individual variables were analyzed using the *t* test, while Person correlation was used to identify the relationship between the studied variables. We used factor analysis (principal component analysis) to establish characteristic of the studied variables. Job satisfaction levels among nursing professionals were determined with 20 questions and factor analysis (principal component analysis) was conducted to reduce the number of variables. In addition, the Kaiser-Meyer-Olkin test and Bartlett’s test were used to assess the appropriateness of using factor analysis and identify job satisfaction factors. For evaluation and examination of the screen chart, we continued to estimate with four factors. The value of Kaiser-Meyer-Olkin test statistics was 0.949, which shows the excellent suitability assessment. Multivariate regression analysis was used to determine the impact of studied independent variables on job satisfaction, and then the proportion of total variation for job satisfaction was explained with the selected independent variables.

## Results

The study included 96 nursing leaders and 413 other employees in nursing. There were 11 men and 498 women. Median age of leaders was 43.5 years (range 33-59) and of other employees 38 years (range 21-60). Leaders spend an average 10.1 years in the leading position, while other employees were employed in the participating hospital for average 16.5 years.

There were significant differences between leaders and employees in ten out of twenty questions on the level of job satisfaction (Table 1). Nurse leaders had significantly higher satisfaction (*t* = 2.946; *P* = 0.003) with the work (mean ± standard deviation, 4.27 ± 0.6), management of the organization (3.41±,0.9 *t* = 2.854; *P* = 0.004), their pay (2.93 ± 1.2, *t* = 2.944; *P* = 0.003), their status in the organization (3.56 ± 0.,9, *t* = 3.981, *P* < 0.001), their motivation for professional development (3.52 ± 0.8; *t* = 3.131; *P* = 0.002), the level of security and reliability of employment (3.94 ± 0.7; *t* = 4.910; *P* < 0.001), the assigned working hours (3.65 ± 0.8; *t* = 3.108; *P* = 0.002), and the ability to participate in the decision-making process (3.51 ± 0.6; *t* = 3.949; *P* < 0.001). Also, nurse leaders had significantly (*t* = 3.237; *P* < 0.001) higher level of job satisfaction (3.49 ± 0.5) than nurses (3.29 ± 0.6).

**Table 1:** Comparison of job satisfaction levels of leaders and employees in nursing

Job satisfaction with	Points on the opinions (N = 509)				T	*P*
	leaders (N = 96)		employees (N = 413)			
	mean ± standard deviation*	standard error of the mean	mean ± standard deviation	standard error of the mean		
The work	4.27 ± 0.6	0.064	4.02 ± 0.7	0.038	2.946	**0.003**
Management of the organization	3.41 ± 0.9	0.090	3.08 ± 1.0	0.051	2.854	**0.004**
Coworkers	3.96 ± 0.6	0.071	3.86 ± 0.8	0.040	1.344	0.180
Interpersonal relations	3.47 ± 0.7	0.074	3.30 ± 0.9	0.045	1.678	0.094
Superior’s leadership style	3.62 ± 0.7	0.077	3.73 ± 0.9	0.046	-1.038	0.300
Provided feedback	3.60 ± 0.9	0.088	3.47 ± 0.8	0.042	1.411	0.159
Opportunities for promotion	3.83 ± 4.2	0.424	3.11 ± 1.0	0.049	1.702	**0.009**
Pay for the work	2.93 ± 1.2	0.119	2.56 ± 1.0	0.054	2.944	**0.003**
One’s status in the organization	3.56 ± 0.9	0.090	3.14 ± 0.9	0.047	3.981	**<0.001**
Motivation for professional development	3.52 ± 0.8	0.086	3.20 ± 0.9	0.045	3.131	**0.002**
Security and reliability of employment	3.94 ± 0.7	0.079	3.49 ± 0.8	0.043	4.910	**<0.001**
The amount of work and the number of assignments	3.32 ± 1.0	0.106	3.36 ± 0.8	0.089	-0.172	0.863
Forms of motivation	3.24 ± 0.9	0.090	3.11 ± 1.0	0.050	1.126	0.261
Working conditions	3.09 ± 1.0	0.106	3.21 ± 1.0	0.050	-0.962	0.336
Education possibilities	3.56 ± 0.8	0.086	3.34 ± 1.0	0.050	2.037	**0.042**
The existing control and penalty system	3.15 ± 0.9	0.097	3.24 ± 0.9	0.044	-0.911	0.363
Working hours	3.65 ± 0.8	0.086	3.33 ± 1.0	0.051	3.108	**0.002**
With concern for employees’ well-being	2.93 ± 0.9	0.100	3.02 ± 1.0	0.054	-0.781	0.435
Praise and the level of trust	3.31 ± 0.8	0.089	3.13 ± 1.0	0.052	1.617	0.107
The ability to participate in the decision-making process	3.51 ± 0.6	0.069	3.18 ± 0.9	0.048	3.949	**<0.001**
**Level of job satisfaction**	3.49 ± 0.5	0.053	3.19 ± 0.6	0.031	3.237	**<0.001**

*Mean (on scale from 1 to 5).

Leaders and employees ranked ten most important factors influencing their job satisfaction and the most important factors were good workplace relationships, followed by pay, praise from the superiors, opportunities for promotion, education possibilities, superiors’ encouragement for work, good working conditions, work responsibility and professional challenges, work-connected freedom and independence, and more free time.

### Correlation analysis of job satisfaction

Correlation analysis was conducted between job satisfaction of nursing leaders and nurses and their age, years of employment in nursing, type of job, level of education, personal characteristics of leaders (integrity, organization, team work, resoluteness, reliability, objectivity, responsibility, confidence, sociability, and ambition), leadership style, and managerial competencies of leaders (vision and goals, communication, conflict solving, motivation, interpersonal relations, team work, problem solving, social authority, delegation, decision making, controlling and introducing change, emotional intelligence, human resource development and quality). There was a slight positive correlation between job satisfaction of nurses and their level of education (r = 0.109; *P* = 0.014), and a negative correlation between job satisfaction and the type of job (r = -0.127; *P* = 0.004), which means that nurse leaders have higher job satisfaction than nurses, and that job satisfaction increases with the level of education. In addition, all examined managerial competencies of leaders (r = 0.626; *P* < 0.001), leadership style (r = 0.514; *P* < 0.001), and personal characteristics of leaders (r = 0.630; *P* < 0.001) positively correlated with job satisfaction of nursing professionals (Table 2).

**Table 2:** Results of Pearson correlation analysis for job satisfaction

	Year of employment	Type of job	Level of education	Personal characteristics of leaders	Leadership style	Managerial competencies
Pearson correlation	-0.006	-0.127*	0.109^†^	0.630*	0.514*	0.626*
Significant (2-tailed)	0.891	0.004	0.014	<0.001	<0.001	<0.001
N	509	509	509	509	509	509

*Correlation is significant at the 0.01 level.

†Correlation is significant at the 0.05 level.

### Factor analysis of job satisfaction

Assessment of job satisfaction was determined with twenty questions. We tried to reduce the number of variables with principal component analysis. The four factors extracted from the principal component analysis for job satisfaction explained 64% of job satisfaction variance (Table 3). The first factor explained as much as 35% of the entire variance; the second factor explained 12%, the third factor 9%, and the fourth factor 8%. We decided to call the first factor motivation and concern for the welfare. In this factor, 9 items were ranked including both material and non-material motivation, with an emphasis on encouragement, praise, trust, control, punishment, and working conditions. The second factor was called leadership style. In this factor, 6 items were ranked including leaders, leadership style, decision making, and feedback. The third factor was called nurses’ professional development. In this factor, 3 items were ranked covering education opportunities, status in the organization, and development. The fourth factor was called cooperation and interpersonal relations. In this factor, 2 items were ranked including relationships with coworkers, leaders, and cooperation between nursing team members.

**Table 3:** Rotated Factor Matrix for four factors of job satisfaction

Job satisfaction with	Factor			
	motivation and concern for the welfare	leadership style	nurses’ professional development	cooperation and interpersonal relations
Concern for employees’ well-being	0.638	0.220	0.398	0.092
Praise with the level of trust	0.626	0.205	0.511	0.073
Forms of motivation	0.574	0.334	0.376	0.192
Working conditions	0.517	0.200	-0.077	0.240
Working hours	0.500	0.311	0.131	0.092
Existing control	0.454	0.217	0.234	0.184
Pay for the work	0.450	0.360	0.198	0.229
The number of assignments	0.420	0.160	0.123	0.125
The work	0.314	0.098	0.004	0.270
Management of the organizational	0.197	0.691	0.213	0.146
Superior’s leadership style	0.341	0.648	0.054	0.036
Provided feedback	0.311	0.627	0.396	0.169
Decision-making process	0.319	0.422	0.313	0.245
Reliability of employment	0.344	0.369	0.121	0.229
Opportunities for promotion	0.097	0.308	0.125	0.009
One’s status in the organization	0.129	0.158	0.750	0.240
Educational possibilities	0.110	0.329	0.680	0.174
Professional development	0.443	0.279	0.510	0.171
Coworkers	0.208	0.012	0.188	0.632
Interpersonal relations	0.179	0.213	0.314	0.614

### Regression analysis of job satisfaction

Independent variables included age of respondents, years of employment at the current hospital, level of education, the readiness of leaders to organize seminars and courses, leadership style, personal characteristics of “good leaders,” and managerial competencies of leaders.

Job satisfaction for nurses in Slovenian hospitals was related to the age of respondents (β = 0.191; *P* = 0.033), number of years they had worked at the current hospital (β = 0.193; *P* = 0.033), selected personal characteristics of leaders (β = 0.158; *P* < 0.001), and managerial competencies of leaders (β = 0.634; *P* < 0.001). These predictors explained 45.7% of variance for the job satisfaction level of nurses (Table 4).

**Table 4:** Regression analysis results for job satisfaction (R^2^ = 0.457)

Characteristics	B*	Standard error	β^†^	*P*
Age (years)	0.013	0.006	0.091	**0.033**
Years of employment at the current hospital	0.011	0.005	0.193	**0.033**
Level of education	0.001	0.024	0.002	0.965
Leaders’ organization of seminars and courses	-0.022	0.054	- 0.020	0.688
Leadership style	-0.185	0.104	- 0.126	0.075
Personal characteristics of leaders	0.159	0.049	0.158	**<0.001**
Managerial competencies of leaders	0.791	0.110	0.634	**<0.001**

*B = unstandardized coefficient.

†β = standardized multiple regression coefficient.

Of the factors included in the research, managerial competencies of leaders had the highest standardized regression coefficient (β = 0.634) and therefore influenced nurses’ job satisfaction most. Finally, we also used a regression equation to determine how selected factors influence the job satisfaction of nurses. The following regression model was made, based on regression analysis results:

Job satisfaction of nurses in participating Slovenian hospitals = a + b1 * age + b2 * years of employment at the current hospital + b3* selected leader characteristics + b4* managerial competencies of leaders

## Discussion

Our research confirmed that job satisfaction of nurses in Slovenian hospitals was at a medium level. Golbasi et al also found a medium satisfaction level in Turkish hospitals (36). Nurses with a higher education have been shown to be more satisfied with their job than those with lower education (37). We found that nurse leaders were more satisfied with their job than other nurses. The lowest levels of satisfaction were shown for pay level, amount of praise and level of trust, involvement in the decision-making process, concern for employees’ well-being, opportunities for promotion, and leadership, and the highest were shown for satisfaction with the job and with coworkers. Similar results were obtained by Sveinsdottir et al (38), who showed that nurses were most satisfied with their coworkers and head nurses, and least satisfied with their opportunities for promotion and pay level. For nursing professionals in Slovenian hospitals, pay level represented the second most important factor of job satisfaction. The pay dimension, which is not a function of organizational structure, was found to limit hospitals in improving nurses’ job satisfaction (39). The factor analysis for job satisfaction in our study yielded four factors, one of which, cooperation and interpersonal relations, explained 8% of the total variance. Nevertheless, respondents expressed a relatively low satisfaction level with personal relationships at their hospital and the leadership style of their immediate superior. Lu et al (2) identified a positive correlation between nurses’ job satisfaction and group cohesion at the workplace. The second factor in our study was leadership style, explaining 12% of total job satisfaction variance. Other studies reported that higher nurse job satisfaction was associated with leadership style focused on people and relationships (40), nurses’ autonomy, control over their practice and nursing leadership on the ward (41), and emotional intelligence of leaders (42). Lorber and Skela Savič (43) found that nurses in Slovenian hospitals wanted to be included in the decision-making process and in setting goals, while Skela Savič and Pagon (44) found that physicians and nurses estimated their level of personal involvement as low and indicated insufficient involvement in work teams. This means that Slovenian hospitals are not taking full advantage of the intellectual capital and experience of their employees.

Four factors extracted from the factor analysis (motivation, leadership style, professional development, and interpersonal relations) explained 64% of the total job satisfaction variance. Another study (34) also obtained four factors (collegial workplace, behavior, relational atmosphere, and outcomes of conflict), which explained 68% of total job satisfaction variance. Ning et al (45), Al-Almeri (4), and Skela Savič et al (20) found that nurses who viewed the working environment as empowering were more likely to provide high quality care, because satisfied employees perform better and are more productive. Enhancing empowerment in a supportive environment would allow nurses to experience satisfaction with their job. Kwak et al (46) also found that management and managerial support had a pronounced effect on nurses’ job satisfaction and the quality of care. Our research confirms that managerial competencies of leaders have the greatest effect on employees’ job satisfaction, explaining as much as 39% of total job satisfaction variance.

Like Sellgren et al (47), we also found that nurses’ job satisfaction correlated positively with leadership style, as well as managerial competencies and personal characteristics of leaders, which explained almost 46% of total job satisfaction variance for nurses. Blegen (11) and Al-Almeri (4) found that job satisfaction of hospital nurses correlated positively with organizational commitment, which explained 41% of variance in job satisfaction.

This study has several limitations. The research framework was based on theoretical findings dealing with leadership style, personal characteristics, managerial competencies of leaders, and the level of nurses’ job satisfaction, so we only studied the influence of some predictors of job satisfaction. The previously tested questionnaire was not used for this research; instead, we prepared most of the items ourselves and tested them before mailing out the questionnaires. The questions were closed-ended and respondents were asked to select one of the provided answers – the disadvantage of this system being a limited number of answers. The questionnaire, too, had certain limitations, such as a relatively long time required for its completion (over 15 minutes). Because the questionnaires were sent by mail and a researcher was not available in participating hospitals, respondents did not get any help in case they did not understand the items and no additional explanations were provided about the content or the manner of completion. Furthermore, the research included a sample of employees from only four Slovenian hospitals, so the data cannot be generalized to the whole population of Slovenian nurses working in hospitals. With constantly changing health system, hospitals will have to recognize the importance of employees’ job satisfaction. One of the key challenges for every organization is to maintain the satisfaction of employees and increase their motivation. This research should be conducted in all health institutions of Slovenia in the next few years; also it would be necessary to constantly monitor job satisfaction of all health care professionals.
